# The effect of theta-burst TMS on cognitive control networks measured with resting state fMRI

**DOI:** 10.3389/fnsys.2013.00124

**Published:** 2013-12-30

**Authors:** Caterina Gratton, Taraz G. Lee, Emi M. Nomura, Mark D'Esposito

**Affiliations:** ^1^Helen Wills Neuroscience Institute, University of CaliforniaBerkeley, CA, USA; ^2^Department of Psychology, University of CaliforniaBerkeley, CA, USA

**Keywords:** fMRI, TMS, cognitive control, functional connectivity, brain networks

## Abstract

It has been proposed that two relatively independent cognitive control networks exist in the brain: the cingulo-opercular network (CO) and the fronto-parietal network (FP). Past work has shown that chronic brain lesions affect these networks independently. It remains unclear, however, how these two networks are affected by acute brain disruptions. To examine this, we conducted a within-subject theta-burst transcranial magnetic stimulation (TBS) experiment in healthy individuals that targeted left anterior insula/frontal operculum (L aI/fO, a region in the CO network), left dorsolateral prefrontal cortex (L dlPFC, a region in the FP network), or left primary somatosensory cortex (L S1, an experimental control region). Functional connectivity (FC) was measured in resting state fMRI scans collected before and after continuous TBS on each day. We found that TBS was accompanied by generalized increases in network connectivity, especially FP network connectivity, after TBS to either region involved in cognitive control. Whole-brain analyses demonstrated that the L dlPFC and L aI/fO showed increased connectivity with regions in frontal, parietal, and cingulate cortex after TBS to either L dlPFC or L aI/fO, but not to L S1. These results suggest that acute disruption by TBS to cognitive control regions causes widespread changes in network connectivity not limited to the targeted networks.

## Introduction

**Figure 1:**
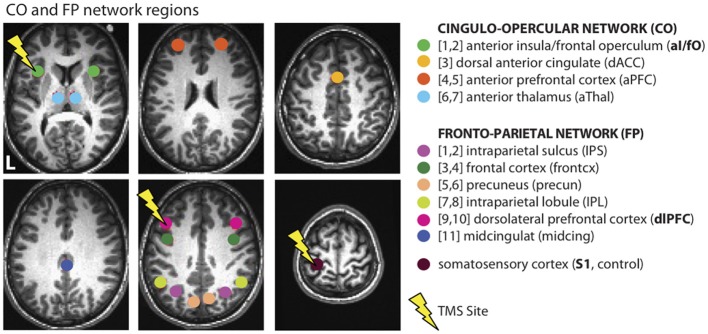
**Cognitive control networks**. Connectivity was examined across and within regions from the CO and FP cognitive control networks, as defined in Dosenbach et al. (2007). These measurements were taken before and after TBS to L dlPFC (a region in the FP network), L aI/fO (a region in the CO network), and L S1 (an experimental control location). Lightning bolts indicate the sites of stimulation. Due to the depth of the targeted aI/fO region, it is likely that stimulation additionally influenced other areas of the overlying frontal operculum that are also part of the CO network (see Figure 2C).

Cognitive control is an essential ability that allows humans to interact with their environment and carry out complex goal-directed behaviors. In the past, it has been suggested that a distributed set of regions may contribute to cognitive control processes (Duncan and Owen, 2000; Koechlin et al., 2003; Dosenbach et al., 2006; Badre and D'esposito, 2007), but it is not clear to what extent these systems operate independently in the brain (see review by Dosenbach et al., 2008). Recent work from Dosenbach and colleagues used activation data from a variety of different tasks (Dosenbach et al., 2006) combined with resting-state functional connectivity (FC) measurements (Dosenbach et al., 2007) to suggest that two separable cognitive control networks exist in the human brain (Dosenbach et al., 2008): the fronto-parietal (or FP) network and the cingulo-opercular (or CO) network (see Figure 1). The FP network is composed of dorsolateral frontal and parietal regions, the precuneus, and the mid-cingulate and is proposed to operate at fast time-scales for moment-to-moment adaptive task control. The CO network is composed of regions in the anterior insula/frontal operculum, anterior prefrontal cortex, dorsal anterior cingulate, and thalamus, and is proposed to operate over longer time-scales for stable task maintenance. In a large population of subjects, these networks clustered separately from one another during rest, suggesting that they may operate in a somewhat segregated fashion (Dosenbach et al., 2007).

More recent data from our laboratory has extended these findings by examining how these networks functionally change following chronic brain damage (Nomura et al., 2010). By examining resting state data from a group of patients who had lesions to these two networks, we demonstrated that damage to one cognitive control network caused changes in functional network interactions that extended throughout the network, beyond the site of structural damage. However, these changes were specific to the impacted network, leaving the other cognitive control network relatively intact. This finding provided strong evidence that these cognitive control networks exhibit independent responses to long-term damage.

However, the patients in the study by Nomura et al. (2010) were all scanned many months (>5 m, on average 8 years) since the incident that caused their lesion, allowing ample time for long-term structural and functional reconfiguration. One previous study examining recovery after stroke to the ventral attention network found that strokes initially impacted widespread regions, causing impairments to both the ventral and dorsal cortical attention networks. However, later during the chronic recovery period, impairments became more selectively limited to the ventral attention network (He et al., 2007). Therefore, it remains an open question how acute disruptions, such as those induced by continuous theta-burst transcranial magnetic stimulation (TBS), will affect the organization of the CO and FP networks. In particular, this may shed light on how these networks interact on a shorter time-scale, such as when both are activated in the service of a cognitive control task, and whether their distinct response to chronic damage represents long-term plasticity or is a signature of disruption and/or rapid functional reorganization that is present immediately after acute interventions.

In this study, we used TBS combined with resting state FC measurements to examine network changes following acute disruption of specific brain areas. In separate sessions, TBS was applied to the left dorsolateral prefrontal cortex (L dlPFC, a region in the FP network), to the left anterior insula/frontal operculum (L aI/fO, a region in the CO network), and to the left primary somatosensory cortex (L S1, an experimental control region that does not participate in either network). Resting state FC was measured before and after TBS for each session. If these networks react independently to acute disruption, as they do to chronic focal brain lesions, then we would expect that TBS to the L aI/fO region will only impact interactions within the CO network and TBS to the L dlPFC region will only impact interactions within the FP network, while TBS to the S1 region will not impact either network. However, it is also possible that the response to acute disruptions will show less independence and reflect more widespread changes that are not limited to the damaged region's network.

## Materials and methods

### Participants

Twenty-seven healthy right-handed participants completed four separate sessions with fMRI and TMS components (11 females, 18–31, mean age = 22.7 years). Participants were not on any antidepressant medications and had no history of epilepsy, anxiety, or neurological disorders. Participants provided written informed consent before the study and were compensated monetarily. The study procedures were approved by the Committee for the Protection of Human Subjects at the University of California, Berkeley.

### Experimental timeline

Each participant completed four combined TMS and fMRI sessions on separate days. During the first day, participants took part in a scanning session during which a structural image, a resting state Echo Planar Imaging (EPI) functional scan, and a perfusion Arterial Spin Labeling (ASL) functional scan were acquired. In addition, resting motor thresholds (see Transcranial Magnetic Stimulation) were measured for each participant during this session.

On the following 3 days, participants began by completing a short EPI and ASL scan. After scanning, they received 40 s of continuous TBS stimulation offline to one of three sites: L dlPFC, L aI/fO, or L S1 (session order was counterbalanced across participants). Participants then re-entered the scanner and completed two more sets of interleaved EPI and ASL scans, resulting in two post-TMS EPI blocks (labeled as post-TMS Block 1 and Block 2 throughout the manuscript) and two post-TMS ASL blocks (not discussed further here). Approximately 10 min elapsed between TBS stimulation and the start of scanning. During scanning, a black fixation cross was presented on a white screen. Participants were instructed to stay still, awake, and with their eyes open; they were told they should allow their mind to wander while trying not to focus on anything in particular. Wakefulness was monitored during all scans using an MR-compatible camera to image the eye. Each session took approximately 1 h to complete. In a few participants, the second ASL scan was not included due to participants either falling asleep (*N* = 2) or the session extending beyond the scanning time limit (*N* = 2).

### Transcranial magnetic stimulation (TMS)

All TMS occurred offline, outside of the scanner. Throughout each stimulation session, participants were seated in a comfortable chair with ear protection. Stimulation was applied using a Magstim figure-of-eight coil (outer winding diameter = 70 mm; *Magstim, UK*). Pulses were delivered with a Magstim rapid stimulator with four booster modules producing biphasic pulses.

On the first day, motor thresholds were calculated using electromyography measured from electrodes on the dominant hand's first interosseous (FDI) muscle. Stimulation was applied over the hand area of the contralateral primary motor cortex, with the coil placed tangentially to the scalp and the handle pointing posteriorly. This area was identified as the area that produced the largest motor evoked potential (MEP) from the FDI when it was relaxed. Each participant's active motor threshold (AMT) was then computed by finding the minimum stimulation intensity at which 5 out of 10 trials produced an MEP in the contralateral FDI while the muscle was contracted at 20% of maximum (electromyographic signal feedback was provided on a monitor to help participant maintain this level of contraction). Past studies have suggested that MEPs show low intra-individual variability across days, but high-inter-individual variability across subjects (Sommer et al., 2002). We therefore collected AMT measurements to reduce variability in stimulation effects across participants, but only measured AMT on 1 day in an effort to reduce scan times in the already long (4 session) experiment.

On the other 3 days, TBS was applied to pre-defined stimulation targets (see Stimulation Sites, below). These targets were localized using a computerized frameless stereotaxic system (*Brainsight software, Rogue Research, Canada*), in which head position is monitored in real time with an infrared camera by using reflective markers placed on the participant's head and the TMS coil. The position of these markers was co-registered to locations on the individual participant's MRI scan that was acquired during the first day. Once the coil was placed over the stimulation target, 40 s of TBS was applied continuously at 80% of AMT. Continuous TBS consists of 50 Hz triplets every 200 ms. This form of TBS has been shown to produce reduced excitability of underlying cortex lasting up to 60 min following stimulation (Huang et al., 2005), allowing for sufficient fMRI recording time.

#### Stimulation sites

Three separate sites were targeted in the three different TBS days: the left dorsolateral prefrontal cortex (L dlPFC), left anterior insula/frontal operculum (L aI/fO), and left primary somatosensory cortex (L S1; sites shown on Figure 1). These regions were defined offline in each individual. The experimental control site, L S1, was defined for each individual as a region on the post-central gyrus 10 mm away from the midline and 5 mm from the top edge of the brain. The other two sites were defined using a procedure that combines individualized anatomical and functional criteria [based on the method from (Eldaief et al., 2011)]. This was done in an effort to optimize the stimulation site for each cognitive control network. Initially, a site was selected in each participant based on coordinates previously published by Dosenbach et al. (2007). These coordinates, along with those from the rest of the regions in the CO and FP networks (shown in Figure 1), were reverse-normalized into native space. For each participant, a CO and FP network map was then created by conducting a whole brain FC analysis using the resting state data from the first baseline day. The time-series was averaged over all regions in the relevant network other than the target site. This averaged time-series was then correlated with every voxel in the brain (see Functional Connectivity for a full description of connectivity methods). For example, a CO whole brain network map would be created by averaging the time-series from all regions in the CO network other than the L aI/fO region and then computing the correlation between this average time-series and every voxel in the brain. Then, within each network map, we found the cluster of connectivity that was closest to the original reverse-normalized spatial coordinates. The peak voxel from this cluster was selected as the stimulation site and used to create a 6 mm spherical region of interest (ROI). If no nearby cluster could be identified (as was the case for 4 subjects for L aI/fO and 2 subjects for L dlPFC), then the original reverse-normalized coordinates were used as the center of the ROI. Note that, although the aI/fO region was deeper on average than the other two sites, a control analysis was conducted to demonstrate that more superficial regions along the aI/fO TMS trajectory had a similar overall pattern of connectivity to the deeper target site (see Functional Connectivity section for a complete description of the control analysis and Results section for the findings). It is likely that stimulation affected processing in both the more superficial frontal operculum along with deeper regions ranging toward the targeted site.

### MRI acquisition parameters

All MRI data were acquired from a 3T Siemens Magnetom Trio scanner using a 12-channel head coil. Structural images were acquired during the first experimental session using an MP FLASH T1-weighted scan. EPI scans were used to measure whole brain Blood Oxygen Level-Dependent (BOLD) signal for resting state FC analysis. EPI scans were obtained using a T2^*^-weighted EPI sequence (*TR* = 2 s, *TE* = 24 ms, flip angle = 60°, in plane matrix = 64 × 64 pixels, each 3.5 × 3.5 mm, with 37 3.5 mm descending axial slices and a 0.7 mm slice gap). A single EPI scan was collected before TBS and two EPI scans were collected after TBS (post-TMS Blocks 1 and 2). Each scan lasted approximately 6 min, during which 180 volumes were acquired. After each EPI scan, 5.5 min ASL scans were collected to measure cerebral perfusion and are not reported in this paper. Note that many of the analyses reported in this paper focus on post-TMS Block 2, which occurred approximately 20 min after TBS, when past experiments would suggest that cTBS effects would be maximal (Hubl et al., 2008); however, results from both blocks are discussed in all sections.

### Data preprocessing

Preprocessing was carried out in AFNI [http://afni.nimh.nih.gov/afni/, (Cox, 1996)]. Images were extracted from DICOMs, slice-time corrected, realigned to the first time point of each session, co-registered to the anatomical image, and smoothed (6 mm FWHM). In addition, the transformation between normal and native space was computed for the anatomical image using SPM segment (Ashburner and Friston, 2005). This was used to reverse normalize published coordinates into native space.

### Functional connectivity

FC was computed using time-series correlations following the procedure described in Fox et al. (2005). Voxel time-series were bandpass filtered (0.009–0.08 Hz). Nuisance signals from white matter, ventricles, and motion along with their temporal derivatives were regressed out [note, however, that the brain's global signal was not included as a regressor due to concerns about biases it could introduce into the correlation structure, e.g., Saad et al., 2012]. These regressors were included in order to reduce noise in the signal from physiological, motion, and scanner artifacts. After nuisance regression, the time-series were averaged across all voxels within an ROI (see Regions of Interest for definitions), and these averaged time-series were used as seeds for FC analyses. In the ROI-to-ROI connectivity analyses, Pearson's time-series correlations were calculated among the averaged time-series from CO and FP network nodes. In the ROI-to-whole brain analyses, Pearson's time-series correlations were calculated between the average time-series of the seeded ROI (L dlPFC and L aI/fO) with each voxel in the rest of the brain. All correlation values were Fisher-transformed before statistical comparison.

In addition, since the aI/fO ROI was deeper, on average, than the other two target regions, we conducted a control analysis to determine whether areas along the aI/fO TMS trajectory that were at the brain surface (and thus closer to the coil and more strongly stimulated by TMS) were also part of the CO network or if their connectivity profile was very different from the target region. In all participants whose aI/fO ROI did not lie directly at the brain surface (*N* = 25), we created a 6 mm spherical region of interest and placed it at the most superficial location along the trajectory of aI/fO stimulation (aI/fO_superficial_). We then calculated the FC between aI/fO_superficial_ and all the regions in the CO and FP networks. Fisher-transformed correlation values were averaged across regions within each network and the resulting aI/fO_superficial_—to—CO and aI/fO_superficial_—to—FP correlation values were directly contrasted in paired *t*-tests.

#### Regions of interest

For the three stimulation sites, regions of interest were defined based on a combination of structural and functional criteria as described under Stimulation Sites. In addition, the other ROIs from the CO and FP networks were defined as a 6 mm sphere centered on the reverse normalized coordinates from Dosenbach et al. (2007) (see Figure 1 for all regions). As described above, an additional ROI (aI/fO_superifical_) was included for a control analysis to determine whether the connectivity profile of sites closer to the TMS coil were similar to the deeper targeted aI/fO ROI. This ROI was created by placing a 6 mm sphere at the most superficial point along the aI/fO TMS trajectory.

#### Statistical analysis and data visualization

ROI-to-ROI analyses within pre-defined networks were compared using ANOVAs on average Fisher-transformed connectivity values within each network. A 3-factor ANOVA was used to examine changes induced by TBS across the entire CO or FP network, with factors of block (pre-TBS, post-TBS Block 1, and post-TBS Block 2), network (CO, FP, and between CO-FP), and TBS-site (aI/fO, dlPFC, and S1). When necessary, *p*-values accompanying the ANOVA were corrected for sphericity using the Huynh-Feldt method [indicated as (HF) in the text]. Significant effects and interactions were further explored with *post-hoc t*-tests. In some cases, a separate ANOVA was conducted for post-TBS block 2, where effects were expected to be maximal (Hubl et al., 2008). In addition, network connectivity to the target nodes alone was separately examined using a 4-factor ANOVA with factors of block (pre-TBS, post-TBS Block 1, post-TBS Block 2), TBS-site (aI/fO, dlPFC, S1), connectivity seed (aI/fO, dlPFC), and target (CO, FP). All reported *t*-tests were paired and accompanied by two-tailed *p*-values. ANOVAs were carried out using the SPSS package (*Version 20, Chicago, IL*). Effect size estimates were provided for ANOVAs (partial-eta squared, η^2^) and for *t*-tests [Cohen's *d*; for paired statistics this was calculated using the standard deviations from each mean and corrected for the correlation between the two means (Morris and Deshon, 2002)].

Network graphs were created using the NetworkX package in python [http://networkx.github.io/]. All other graphs and statistical analyses were created using python's matplotlib [http://matplotlib.org/] and scipy [http://www.scipy.org/] packages. Voxel-to-whole brain analyses were corrected for multiple comparisons using AFNI's 3dClustSim, which uses Monte Carlo simulations on *p* < 0.001 uncorrected maps to estimate the minimum cluster size that would be significant [i.e., such that *p*(corrected) < 0.05], based on the size of the whole-brain volume and the amount of smoothness in the data.

## Results

### Identification of CO and FP networks within individuals before TBS

**Figure 2:**
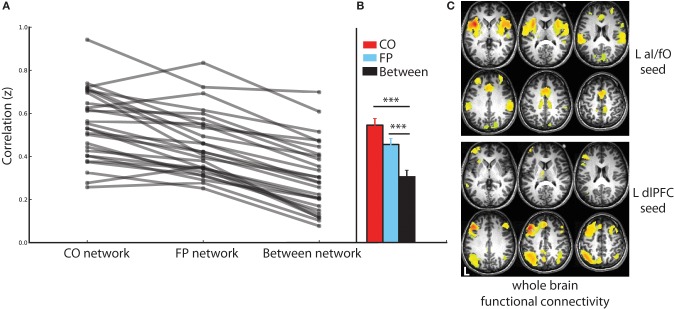
**Connectivity in the CO and FP networks before TBS. (A)** Connectivity within the CO and FP networks and across the two networks are shown for each individual (gray lines). Across all individuals, within network connectivity was higher than between network connectivity. **(B)** Statistics across the group of subjects confirm these findings, with connectivity within the CO network (red) and within the FP network (blue) significantly higher than connectivity across the two networks (black; ^***^p < 0.001). **(C)** Whole brain functional connectivity analyses seeded in the locations targeted for TBS recapitulated the CO and FP networks: CO network regions were functionally connected with the L aI/fO region (top), and FP network regions were functionally connected with the L dlPFC region (bottom; in this and subsequent figures L marks the left hemisphere of the brain).

Using the resting-state fMRI data collected before TBS, the CO and FP networks were dissociable in each individual subject (see Figure 2). ROI-to-ROI connectivity analyses demonstrated that within network connectivity was significantly higher than between network connectivity for both the CO network [*t*_(26)_ = 15.16, *p* < 10^−13^, *d* = 2.920] and the FP network [*t*_(26)_ = 13.53, *p* < 10^−12^, *d* = 2.659] and that this pattern was evident across all individual subjects (Figures 2A,B). Furthermore, seeded whole brain analyses also produced a similar result: seeding either L aI/fO or L dlPFC (the regions that were subsequently stimulated) produced a map of regions that recapitulated, respectively, the CO and FP networks {see Figure 2C, displayed at [*t*_(26)_ > 10.29, *p*(uncorr) < 10^−10^, *p*(FDR corrected) < 10^−5^]}. In addition, even an ROI placed at the most superficial location along the trajectory of aI/fO stimulation (aI/fO_superficial_) was still more highly correlated with the CO network than the FP network [*t*_(24)_ = −2.09, *p* < 0.05, *d* = 0.445; this analysis excluded the 2 participants whose original aI/fO ROI already was already located on the brain surface]. These results suggest that, prior to stimulation, the targeted regions were integrated within functionally correlated and dissociable networks.

### The effect of TBS on CO and FP networks

**Figure 3:**
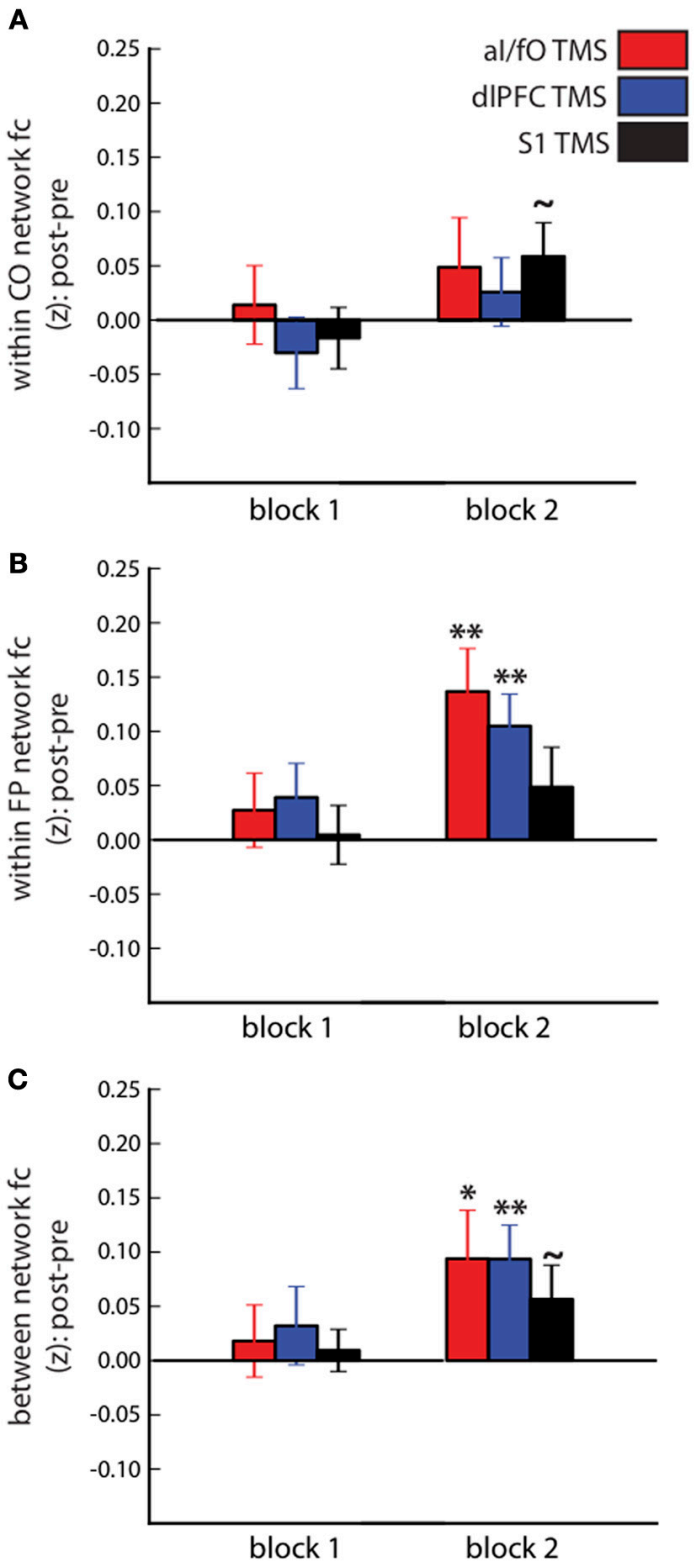
**Connectivity within and between the CO and FP networks after TBS**. Changes in connectivity after TBS (post-TBS Block 1 or 2 minus pre-TBS) are shown for the CO network **(A)**, FP network **(B)**, and between the two networks **(C)**. Different sites of TBS (red, aI/fO; blue, dlPFC; black, S1) did not have systematically different effects on within- and between-network connectivity, although connectivity generally tended to increase after TBS (Post-TBS vs. Pre-TBS: ^**^p < 0.01, ^*^p < 0.05, ^~^*p* < 0.10).

The influence of TBS on the average connectivity of each network was measured with a three-way ANOVA, with factors of block (pre-TBS, post-TBS Block 1, and post-TBS Block 2), network (CO, FP, and CO-FP), and TBS site (aI/fO, dlPFC, and S1), after averaging over all pairwise connections within the CO network, within the FP network, or across the two networks (see Figure 3). This ANOVA revealed a trend toward an interaction between block, network, and TBS site [*F*_(8, 208)_ = 2.115, *p*(HF) = 0.064, η^2^ = 0.075] suggesting that TBS has weak selective effects on different ROIs. In addition, focusing on post-TBS Block 2 [which occurred approximately 20 min after TBS, when past findings would suggest stronger effects on fMRI signals (Hubl et al., 2008)], we also found a trend for a significant three-way interaction [*F*_(4, 109)_ = 2.47, *p*(HF) = 0.074, η^2^ = 0.087]. These results seem to be mainly driven by increases in FP network FC after TBS to either cognitive control site [aI/fO TBS: *t*_(26)_ = 3.45, *p* < 0.01, *d* = 0.697; dlPFC TBS: *t*_(26)_ = 3.57, *p* < 0.01, *d* = 0.690]. However, TBS to either of these sites did not yield a significantly greater increase in FP network connectivity than TBS to S1 [aI/fO TBS vs. S1 TBS: *t*_(26)_ = 1.65, *p* = 0.11; dlPFC TBS vs. S1 TBS: *t*_(26)_ = 1.24, *p* = 0.23]. In addition, TBS to each of the three sites caused an increase in connectivity between the CO and FP networks [aI/fO TBS: *t*_(26)_ = 2.11, *p* < 0.05, *d* = 0.412; dlPFC TBS: *t*_(26)_ = 2.98, *p* < 0.01, *d* = 0.573; S1 TBS: *t*_(26)_ = 1.78, *p* = 0.084, *d* = 0.346].

Network connectivity measures increased significantly over time, regardless of the site of TBS [main effect of block: *F*_(2, 52)_ = 8.60, *p* < 0.001, η^2^ = 0.249]. There was also an interaction between block and network [*F*_(4, 104)_ = 3.251, *p*(HF) < 0.03, η^2^ = 0.111], which was driven by larger increases in connectivity within the FP network and between the FP and CO network, compared with the connectivity within the CO network [average change in FC after TBS, FP vs. CO: *t*_(26)_ = 2.45, *p* < 0.03, *d* = 0.471; FP-CO vs. CO: *t*_(26)_ = 2.65, *p* < 0.02, *d* = 0.510]. Finally, a large main effect of network connectivity magnitude [*F*_(2, 52)_ = 128.2, *p* < 10^−20^, η^2^ = 0.831] was found, recapitulating the persistence of stronger within- than between-network connectivity across the entire fMRI session [within CO vs. between network connectivity: *t*_(26)_ = 17.62, *p* < 10^−15^, *d* = 3.398; within FP vs. between network connectivity: *t*_(26)_ = 13.01, *p* < 10^−12^, *d* = 2.511].

**Figure 4:**
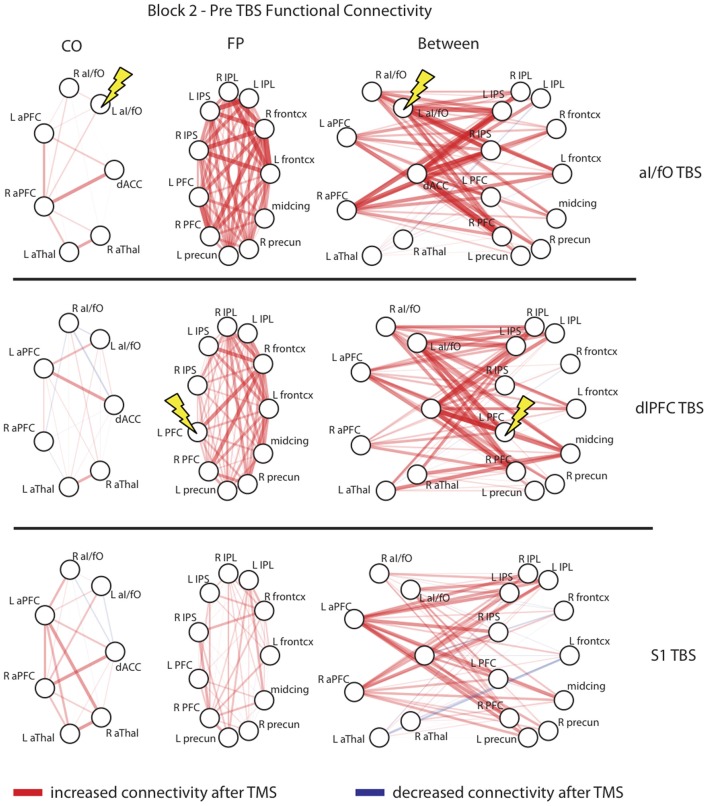
**Network interactions after TBS**. Connectivity changes in the second block after TBS are shown for each individual connection with the CO network (**Left column**), the FP network (**Middle column**), and between the two networks (**Right column**) after TBS to the L aI/fO (**Top row**), L dlPFC (**Middle row**), and L S1 (**Bottom row**). As can be seen, TBS generally increased connectivity across all nodes of the network, with no unique effects seen at the stimulation site. Edge color represents the sign of change after TBS (red, increases; blue, decreases) and the line thickness and opacity indicates the magnitude of change. Lightning bolts highlight the region targeted in each row.

Figure 4 plots the changes for every ROI to ROI connection within or across the CO and FP networks in post-TBS Block 2. The changes summarized in Figure 3 did not appear to be specific to any single connection in these networks, but rather seem to be generally present across all network connections. In particular, in analyses restricted to examining connectivity of only the stimulated ROI to other ROIs in the CO and FP networks, rather than summarizing connectivity across the entire network, the pattern was in the same direction as the network-wide effects but did not reach significance [ANOVA with factors of block (pre, post-TBS Block 1, or post-TBS Block 2), TBS site (aI/fO, dlPFC, or S1), seed (aI/fO or dlPFC), and network (CO, FP), interaction between block × TBS site × seed × network: *F*_(4, 104)_ = 1.45, *p*(HF) = 0.23]. It therefore appears as though focal acute disruptions from TBS are causing widespread, non-specific effects, with generalized increases in connectivity, particularly in connectivity within the FP network after TBS to either cognitive control network region.

### The effects of TBS across the whole brain

**Figure 5:**
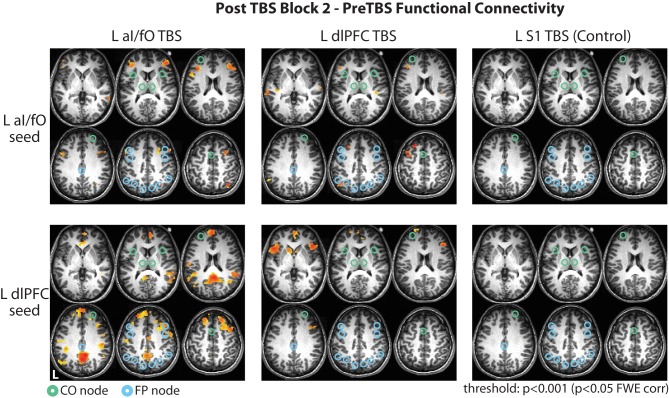
**Whole brain changes in connectivity after TBS**. Changes in connectivity of the L aI/fO region (**Top row**) and L dlPFC region (**Bottom row**) to the whole brain in the second block after TBS are shown following TBS to L aI/fO (**Left column**), L dlPFC (**Middle column**), and L S1 (**Right column**). TBS to L aI/fO and L dlPFC led to widespread increases in connectivity across lateral frontal, parietal, and cingulate regions (see Tables 1, 2). No significant changes were seen after TBS to L S1. Connectivity values are thresholded at *p* < 0.001 (*p* < 0.05 FWE cluster correction; red values indicate increases in connectivity after TBS). Nodes from the CO and FP networks are overlaid on the brain maps in green and cyan, respectively.

In a final exploratory analysis we examined the changes in connectivity of the L aI/fO and L dlPFC seed to the whole brain, after TBS to any of the three targeted locations (see Figure 5). Here we focus again on the second block after TBS (no significant cortical clusters were observed during the first block at *p*[corr] < 0.05, minimum cluster size threshold range = 56–61 voxels). During this time window, TBS to either of the cognitive control network sites caused widespread increases in the connectivity of the aI/fO and dlPFC seeds to lateral frontal, parietal, and cingulate cortices, not limited to regions within the CO and FP network ROIs [*p*(corr) < 0.05, minimum cluster size threshold range = 59–72 voxels].

### aI/fO stimulation

TBS to L aI/fO led to increases in connectivity of L aI/fO, with the largest clusters in bilateral regions of lateral inferior and middle frontal gyrus (including clusters near the dlPFC site of the FP network) and a cluster in the right intraparietal sulcus (proximal to the IPS node of the FP network). In addition, there were smaller clusters in the right superior frontal gyrus, orbitofrontal cortex, posterior temporal lobe, and left anterior temporal lobe. TBS to aI/fO also increased connectivity of the L dlPFC, primarily with regions in the anterior and posterior cingulate cortex, bilateral supramarginal/angular gyrus [together these sites are often characterized as part of the default mode network (Raichle et al., 2001; Fox et al., 2005; Buckner et al., 2008)], and the bilateral superior frontal gyrus. Small clusters of increased connectivity were also seen in left precentral gyrus, the right inferior anterior insula, and the midcingulate (see Table 1 for all clusters).

**Table 1:** **Clusters showing increased connectivity after L aI/fO TBS**.

**Clusters**	**Peak coordinate (MNI, LPI)**	**Size (voxel, 2 mm^3^)**
**aI/fO connectivity**		
R inferior frontal sulcus/middle frontal gyrus	+40.0 +38.0 +14.0	638
L inferior frontal sulcus/middle frontal gyrus	−40.0 +32.0 +14.0	368
L posterior inferior frontal sulcus/middle frontal gyrus	−46.0 +8.0 +36.0	266
R intraparietal sulcus	+38.0 −62.0 +52.0	187
R superior frontal gyrus	+30.0 +16.0 +46.0	149
R lateral orbitofrontal cortex	+34.0 +38.0 −10.0	142
L rostral superior temporal gyrus	−54.0 −6.0 −10.0	84
R middle temporal gyrus	+64.0 −46.0 +0.0	72
**dlPFC connectivity**		
Posterior cingulate cortex	+8.0 −50.0 +30.0	2008
R superior frontal gyrus	+42.0 +14.0 +52.0	1737
L rostral superior frontal gyrus	−22.0 +46.0 +44.0	1416
Anterior cingulate cortex	−4.0 +52.0 −8.0	1055
R supramarginal gyrus/angular gyrus	+62.0 −46.0 +22.0	637
L supramarginal gyrus/angular gyrus	−44.0 −42.0 +22.0	260
Rostral precuneus	−0.0 −48.0 +64.0	239
R anterior supramarginal gyrus	+50.0 −24.0 +30.0	179
L precentral gyrus	−60.0 −20.0 +20.0	163
L posterior angular gyrus	−54.0 −68.0 +24.0	150
R anterior middle temporal gyrus	+66.0 −12.0 −8.0	117
R inferior anterior insula	+32.0 +22.0 −16.0	102
L angular gyrus	−58.0 −54.0 +16.0	90
L caudal middle frontal gyrus	−36.0 +26.0 +44.0	82
Midcingulate gyrus	+4.0 −8.0 +38.0	75

*This table lists all clusters that showed significant changes in connectivity in the second block after L aI/fO TBS (p < 0.001 uncorrected, p < 0.05 FWE cluster-size corrected). The top half of the table lists regions that exhibited changes in connectivity with L aI/fO, and the bottom half of the table lists regions that exhibited changes in connectivity with L dlPFC. No significant clusters showed decreased connectivity after L aI/fO TBS*.

### dlPFC stimulation

TBS to L dlPFC, on the other hand, caused increases in connectivity of the L aI/fO with a few clusters in the left dorsal premotor cortex, middle and superior frontal gyrus, and angular gyrus. Smaller clusters of connectivity increases were also found in the left middle and superior temporal gyri and the right supramarginal gyrus. L dlPFC increased in connectivity with bilateral regions of the anterior insula/inferior frontal gyrus (near the aI/fO node of the CO network), anterior cingulate, and medial superior frontal gyrus. In addition, small increases were seen in the right middle frontal gyrus and anterior superior frontal gyrus (see Table 2 for all clusters).

**Table 2:** **Clusters showing increased connectivity after L dlPFC TBS**.

**Clusters**	**Peak coordinate (MNI, LPI)**	**Size (voxels, 2 mm^3^)**
**aI/fO connectivity**		
L angular gyrus/intraparietal sulcus	−48.0 −70.0 +36.0	160
L dorsal premotor cortex	−42.0 +12.0 +44.0	144
L middle frontal gyrus	−42.0 +26.0 +24.0	135
L superior frontal gyrus	−24.0 +30.0 +48.0	135
L middle temporal gyrus	−64.0 −34.0 +4.0	101
R supramarginal gyrus	+44.0 −32.0 +18.0	69
L superior temporal gyrus	−42.0 −24.0 +0.0	64
**dlPFC connectivity**		
L anterior insula/inferior frontal gyrus	−46.0 +18.0 −4.0	1024
R anterior insula/inferior frontal gyrus	+44.0 +24.0 +0.0	535
Medial superior frontal gyrus	−0.0 +6.0 +64.0	246
Anterior cingulate	+2.0 +58.0 +4.0	237
R middle frontal gyrus	+46.0 +28.0 +22.0	208
L anterior superior frontal gyrus	−20.0 +60.0 +10.0	91

*This table lists all clusters that exhibited significant changes in connectivity in the second block after L dlPFC TBS (p < 0.001 uncorrected, p < 0.05 FWE cluster-size corrected). The same conventions are used in this Table as in Table 1. No significant clusters showed decreased connectivity after L dlPFC TBS*.

### S1 stimulation

TBS to S1 did not cause any significant changes in connectivity across the whole brain, suggesting that these widespread effects may be somewhat specific to stimulation of cognitive control network regions (Figure 5).

### Comparison between stimulation conditions

Direct comparisons between aI/fO TBS and S1 TBS at the whole brain level [*p*(uncorr) < 0.001, *p*(corr) < 0.05] showed that aI/fO TBS increased connectivity between dlPFC and default mode regions significantly more than S1 TBS (with significant clusters in the posterior cingulate, anterior cingulate, angular gyrus, and anterior temporal cortex, along with a small region in the frontal cortex that showed relatively decreased connectivity after aI/fO TBS; see Table 3). No other significant effects were seen at the whole-brain level for direct comparisons between either aI/fO TBS or dlPFC TBS and S1 TBS.

**Table 3:** **Clusters showing significantly different changes in connectivity after TBS to different sites**.

	**Clusters**	**Peak coordinate (MNI, LPI)**	**Size (voxels, 2 mm^3^)**
**aI/fO vs. S1 TBS**			
aI/fO connectivity	^*^R middle frontal gyrus	+40.0 +46.0 +14.0	266
	^*^L caudal middle frontal gyrus	−50.0 +6.0 +36.0	244
	^*^R lateral orbitofrontal cortex	+32.0 +46.0 −8.0	236
	^*^L middle frontal gyrus	−38.0 +32.0 +14.0	210
	^*^Bilateral caudate nucleus	−12.0 −2.0 −10.0	208
	^*^R caudal middle frontal gyrus	+54.0 +18.0 +36.0	208
dlPFC connectivity	Posterior cingulate cortex	−2.0 −50.0 +34.0	415
			
	R anterior middle temporal gyrus	+66.0 −14.0 −8.0	246
	Rostral anterior cingulate cortex	−4.0 +52.0 −10.0	195
	Anterior cingulate cortex	−8.0 +26.0 −4.0	90
	L angular gyrus	−54.0 −66.0 +30.0	75
	Anterior cingulate cortex	+4.0 +30.0 −18.0	67
	R anterior middle frontal gyrus, white matter (*negative*)	+26.0 +42.0, +2.0	62
**dlPFC vs. S1 TBS**			
aI/fO connectivity	None		
			
dlPFC connectivity	^*^L anterior insula/inferior frontal gyrus	−52.0 +22.0 −2.0	398
			
	^*^R superior temporal sulcus	+54.0 −10.0 −8.0	300
	^*^R temporal pole	+48.0 +24.0 −26.0	291

*This table lists clusters that exhibited significantly different changes in connectivity between the different TBS conditions in the second block after TBS. The first section of the table lists significant clusters in the comparison between aI/fO TBS and S1 TBS, and the second section lists significant clusters in the comparison between dlPFC and S1 TBS. No significant differences were seen in the direct comparisons between aI/fO and dlPFC TBS. In each section, changes in connectivity with the aI/fO ROI are listed first, followed by changes in connectivity with the dlPFC ROI*.

* *Alternative threshold: p(uncorrected) < 0.01, p(FWE corrected) < 0.05*.

However, if a more relaxed uncorrected threshold was used [*p*(uncorrected) < 0.01, *p*(FWE corrected) < 0.05, minimum cluster sizes 186–192 voxels], then aI/fO TBS showed significantly greater increases in connectivity than S1 TBS between aI/fO and several clusters of the bilateral middle frontal gyrus (along with a small subcortical cluster near the caudate nucleus). Also, at this threshold, dlPFC TBS caused significantly greater increases in connectivity than S1 TBS between dlPFC and a large cluster in the left anterior insula, along with clusters in the right temporal lobe. Similar clusters to these were also found at slightly different uncorrected thresholds [*p*(uncorr) < 0.005, *p*(corr) < 0.05] and at the standard uncorrected threshold when using the average of both post-TBS blocks, suggesting that they may represent stable regions of increases in connectivity that are slightly weaker in magnitude and therefore not detected at the standard uncorrected threshold in block 2. See Table 3 for a full list of clusters surviving direct comparisons. No clusters reached significance in the direct comparisons between TBS to aI/fO and TBS to dlPFC (even at the alternative thresholds used above).

### Summary

Intriguingly, both the L aI/fO and, especially, the L dlPFC showed enhanced connectivity with regions very proximally located to sites in the opposing network (FP for L aI/fO and CO for L dlPFC) when TBS was applied to those regions. This may suggest that TBS is increasing connectivity between the two networks in regions that are not completely captured by the ROI-to-ROI analysis previously conducted. In addition, TBS to aI/fO caused a strong increase of the coupling between dlPFC and regions associated with the default mode network. No significant changes were seen after S1 TBS, and the effects of aI/fO TBS and dlPFC TBS were seen in direct contrasts with S1 TBS, although not always at standard uncorrected thresholds. No clusters survived direct comparisons between aI/fO and dlPFC TBS, suggesting that, although TBS caused widespread changes in the connectivity of these regions, the profiles of connectivity changes did not differ dramatically between the two regions.

## Discussion

Here we have shown that TBS to a node in either the CO or FP network causes non-specific changes in network connectivity both between and within the CO and FP networks. ROI-to-ROI analyses did not reveal any changes based on cognitive-control site TBS that were significantly different from TBS to S1, and generalized changes such as these may have a number of potential interpretations (see section below). However, ROI-to-voxel analyses revealed that many different regions in frontal, parietal, and cingulate cortex showed increased connectivity after TBS to aI/fO (in the CO network) or dlPFC (in the FP network) which was not observed after TBS to S1. This suggests that acute disruption to cognitive control networks causes widespread changes in network connectivity that are not confined to the networks themselves.

### Acute and chronic changes in network interactions after nodal disruption

Many previous studies have suggested that focal damage can modify FC across regions remote from the lesion site and throughout distributed networks (He et al., 2007; Grefkes et al., 2008; Mintzopoulos et al., 2009; Sharma et al., 2009; Warren et al., 2009; Carter et al., 2010; Nomura et al., 2010; Van Meer et al., 2010; Wang et al., 2010; Ovadia-Caro et al., 2013). Moreover, after damage, changes in behavioral recovery over time track with changes in FC in particular subsystems [attention: (He et al., 2007; Carter et al., 2010), somatomotor: (Sharma et al., 2009; Carter et al., 2010; Wang et al., 2010), somatomotor in rats: (Van Meer et al., 2010), multiple cortical networks: (Ovadia-Caro et al., 2013)], suggesting that modified connectivity may serve as a sensitive marker (and perhaps one of the driving mechanisms) of reorganization after disruption.

However, although direct comparisons are unfeasible, the pattern of network changes observed here following TBS in healthy individuals appear distinct from those reported in patients with focal brain lesions to either the CO or FP networks (Nomura et al., 2010). First, in this past study of lesion patients, increasing amounts of chronic damage to the CO network was related to decreases in the magnitude of connections among intact CO network nodes but unrelated to connectivity in the FP network. Similarly, increasing amounts of chronic damage to the FP network was related to decreased magnitude of connectivity among intact FP network nodes but not the CO network. In the present study, however, TBS in healthy individuals to either cognitive control region increased the connectivity within the FP network (along with connectivity between the two networks) and across a widespread set of regions not confined to either network. Apparent differences in the results from these two studies may in part be due to the amount of time between disruption of nodal function and scanning (short in the case of TBS and long-term in the case of the focal brain lesions, e.g., greater than 6 months). Previous work examining recovery of the dorsal and ventral attention networks after strokes that impacted the ventral attention network reported disruption of the connectivity of both networks during acute periods soon after an infarct (He et al., 2007). However, later in recovery, disruptions became more selectively limited to the ventral attention network. These results mirror the selective nature of disruptions seen in chronic lesion patients (Nomura et al., 2010) and the diffuse, non-selective effects of disruptions seen here acutely after TBS.

Furthermore, although it initially may appear counter-intuitive that the general pattern of effects in this study would be an increase in connectivity after TBS rather than a decrease, given the large amount of behavioral and physiological evidence suggesting that continuous TBS has inhibitory effects on local neural processing [reviewed in (Hoogendam et al., 2010; Thut and Pascual-Leone, 2010; Cardenas-Morales et al., 2011)], this seemingly inverted direction of effects has been seen in a number of other studies. Excitatory TMS has been associated predominantly with decreased connectivity (Eldaief et al., 2011; Bilek et al., 2013), studies that used inhibitory 1 Hz TMS have found remote increases in connectivity (Vercammen et al., 2010; Eldaief et al., 2011), and one study which used both excitatory and inhibitory quadripulse TMS saw inverse changes in connectivity for both [increased connectivity after inhibitory TMS and decreased connectivity after excitatory TMS (Watanabe et al., 2013); see (Fox et al., 2012a) for a review of connectivity changes in fMRI-TMS studies]. One possibility is that our findings and the others described here reflect compensatory mechanisms in network activity following TMS, in this case up-regulating distributed activity after decreases in local activity caused by TBS.

Furthermore, the vast majority of evidence suggesting that TBS decreases local activity stems from studies that have examined changes occurring after TBS to motor or sensory cortex; it thus remains an open question whether TBS has similar effects and mechanisms over all other areas of cortex. In addition, the effects of TMS can vary due to a number of reasons [including previous activity, age, time-of-day, gender, presence of specific genetic markers, etc. (Ridding and Ziemann, 2010)] and even reverse in some cases of motor cortex TBS when participants are asked to contract muscles shortly before or after stimulation (Gentner et al., 2008; Huang et al., 2008). Therefore, given the variability in TMS effects and our limited knowledge of the physiological consequences of TBS, any mechanistic interpretations of the changes observed in our study remain speculative.

Moreover, a mixed pattern of bidirectional changes in FC following acute disruption is also present in past studies of patients with focal lesions. Although most studies have suggested that FC predominantly decreases relative to matched controls (He et al., 2007; Nomura et al., 2010), some studies have demonstrated that the recovery period after damage is characterized by both increases and decreases in connectivity, depending on the connection examined (Mintzopoulos et al., 2009; Wang et al., 2010; Park et al., 2011). In addition, experimental strokes induced in rats, although generally causing decreases in inter-hemispheric connectivity, also led to some increases in FC within the contralesional hemisphere (Van Meer et al., 2010). These results suggest that disruption and recovery can lead to a varied pattern of changes that involve a mixture of up- and down-regulation of connectivity over time.

### Increases in near-cross-network coupling seen in whole brain analysis

The changes after TBS in our study were better captured by our whole-brain analysis than the ROI-to-ROI analysis. In fact, the whole-brain analysis suggested that there were increases in “near-cross-network” coupling in regions adjacent to the examined ROIs that were not evident in ROI-based analysis. For example, TBS to dlPFC increased the connectivity between dlPFC and a region in the anterior insula/frontal operculum that was ventral to the aI/fO ROI defined a priori in the CO network. Similarly, TBS to aI/fO led to increases in connectivity to sites in the middle frontal gyrus and posterior parietal cortex that were near the dlPFC and IPS regions, respectively, of the FP network.

These findings may imply that the ROIs used in our ROI-to-ROI analysis were non-optimal and did not effectively capture the CO and FP networks. However, these same ROIs during pre-TBS periods revealed the CO and FP networks (as indicated by higher within- than between-network connectivity and seed-based analyses) in every individual. This suggests that our findings are not due to selection of poor ROIs. One possibility is that this extended connectivity following TBS reflects additional/expanded regions in the CO and FP network. These may include regions that normally participate in these networks (but are outside of the arbitrarily sized spherical ROI) or regions that normally exhibit sub-threshold/poorer network allegiance. A comparison of the patterns of connectivity before TBS shown in Figure 2C to the regions showing enhanced connectivity after TBS in Figure 5 supports this former notion: baseline network connectivity extends into the sites of post-TBS near-cross-network coupling. Furthermore, one past study has suggested that there may be gradients in the connectivity patterns within the broad extent of regions classically defined as components of the frontoparietal control and default mode networks and that these gradients vary depending on participants' age (Anderson et al., 2011). It is possible, therefore, that TBS may act as another factor that operates on the steepness of these gradients, thus effectively expanding the region of the ROI by increasing the strength of connectivity with other (normally weaker) regions in the near vicinity.

An increase in cross-network coupling may also indicate that additional regions in the opposing network are recruited in a compensatory fashion after TBS. Cooperation between these two normally independent networks may be necessary to maintain function in the acute phase of nodal disruption. Alternatively, increased inter-connectivity among these networks may be a sign of network dysfunction resulting from TBS, whereby these networks are no longer able to sufficiently suppress interactions among one another. Further studies, with behavioral measures associated with each of these networks, would help to tease apart these possibilities.

### aI/fO TBS increased the coupling between dlPFC and regions in the default mode network

In addition to an increase in near-cross-network coupling, TBS to aI/fO caused dlPFC to become more tightly coupled with nodes in the default mode network (DMN), such as the posterior and anterior cingulate and angular gyrus (Buckner et al., 2008). Interestingly, TBS to dlPFC only produced relatively small changes in the connectivity of aI/fO that were not significantly different from changes induced by S1 TBS. This may suggest a hierarchy to the relationship between these two networks, with TBS to CO network regions able to more strongly influence FP (and default mode) network regions than the reverse (at least during the acute period after TBS).

In the past, regions in extended frontoparietal cognitive control networks have been found to couple dynamically with the DMN under particular task demands (Spreng et al., 2010) or in a manner that was correlated with individual differences in dopamine levels (Dang et al., 2012) and age (Spreng and Schacter, 2012). Recent studies have suggested that aI/fO may act as a critical node that mediates the relationship between remote regions in the dorsal frontoparietal cognitive control network and the default mode network. In particular, Sridharan et al. (2008) found that the anterior insula causally influenced connectivity of the DMN and a “central executive network” (a dorsal frontoparietal cognitive control network similar to the FP network) in the context of three separate tasks. These finding prompted the creation of a triple-network model (Menon and Uddin, 2010; Menon, 2011) that proposes a hierarchical relationship among these networks, with the salience network (similar to the CO network, with a prominent node in the anterior insula) causally influencing the DMN and the central executive network. In support of this model, recent findings in patients with schizophrenia (Manoliu et al., 2013), frontotemporal dementia (Chiong et al., 2013), and traumatic brain injury (Bonnelle et al., 2012) all suggest that the anterior insula (or broader salience network) influences DMN interactions. Within this context, then, our findings support the idea that TBS to aI/fO is another type of perturbation of the system that can cause dynamic changes in the coupling between the FP and default mode networks.

Another, not mutually exclusive, possibility is that changes in connectivity found following aI/fO and dlPFC TBS may be driven by the different network-level roles these regions play within the CO and FP networks, respectively. Damage to regions with many between-network connections (connectors) has been shown in the past to cause more widespread impacts on a variety of different networks, whereas this is not true of damage to regions with many within-network connections [within-module hubs; e.g., (Gratton et al., 2012)]. If the aI/fO region acts as more of a connector region, linking the CO to other networks, and the dlPFC region acts as more of a within-network hub node with links predominantly to other areas in its own network, this might explain why damage to aI/fO would have relatively greater impact on FP network connectivity than damage to dlPFC has on CO network connectivity.

### Generalized increases in connectivity after TBS

Regardless of the site of TBS (dlPFC, aI/fO, or S1), the ROI-to-ROI analysis revealed increases in connectivity both within and between the two cognitive control networks. These increases were significantly higher in FP and between network connectivity than CO network connectivity. These non-specific effects point to the clear need for other TBS locations to act as experimental control stimulation sites in studies seeking to make strong statements regarding the specificity of TBS effects. The interpretation of generalized changes such as these is difficult, as they may be due to true but widespread effects of TBS, non-neural changes from TBS stimulation, or other variables such as drowsiness and arousal that may change over time. Past studies of dynamic connectivity have also reported increased network activity throughout scan sessions (Allen et al., 2012), and studies of sleep/drowsiness have also suggested that network connectivity may generally increase across the recording period (Fukunaga et al., 2006).

In particular, only a few prior studies have examined changes in FC during resting state fMRI after TMS, and none of these studies have used different stimulation sites to examine the specificity of their findings to the stimulation location (Van Meer et al., 2010; Vercammen et al., 2010; Eldaief et al., 2011). One study applied 1 Hz TMS over dlPFC and observed decreases in resting state connectivity between the DMN and the lateral temporal cortex compared with sham stimulation, but did not examine other sites or networks to determine whether this effect was selective to the particular stimulation condition (Van Der Werf et al., 2010). Another recent study stimulated a node in the default mode network with either 1 or 20 Hz TMS and observed either increases (for 1 Hz TMS) or decreases (for 20 Hz TMS) in connectivity across default mode locations. This study controlled for both the specificity of effects to stimulation frequency (note again the inverted direction of effects in this study) and network examined, but did not test for the specificity in stimulation site (Eldaief et al., 2011). Therefore, our study design is unique in that it allows us to assess specificity in stimulation site effects on resting state connectivity. Critically, this allowed us to observe the different effects (or, for certain contrasts, the lack thereof) that result from stimulating distinct, putatively independent, networks and experimental control locations.

### Methodological limitations

Several difficulties arise in conducting a combined resting state fMRI—TBS study. First, a large amount of variability exists in TMS effects across individuals, and these may have a wide variety of causes [e.g., previous activity, differences in specific genetic markers, time-of-day, gender, age, etc.; see review by (Ridding and Ziemann, 2010)]. Here, we used a large sample size (relative to other TBS studies) and a within-subjects design to mitigate these effects.

Second, differences may exist in the exact functional/ anatomical locations of stimulation sites across individuals that may contribute to variability that is often seen in TBS effects. Here, we used a method adapted from that of Eldaief et al. (2011) to target subject-specific network regions (see also Fox et al., 2012b). In support of the success of this method, we found that before TBS, the proposed networks were functionally connected with the target sites in all individuals and dissociable from one another. This suggests that variability in these effects is not driven by imprecise targeting of stimulation sites across individuals.

Third, although TMS is often characterized as a method for creating temporary “virtual lesions” (e.g., Pascual-Leone et al., 1999), TMS and chronic focal lesions differ along a number of dimensions. Whereas TBS is fairly selective in the location that is targeted (though see additional discussion on TBS selectivity below), the size and location of lesions after brain injury differ across individuals and is not under experimental control. However, in our previous lesion study (Nomura et al., 2010), the most common locations for lesions overlapped with aI/fO and dlPFC regions, thus increasing the similarity between the acute and chronic approaches in this regard. In addition, past studies have suggested that TBS impacts underlying cortical activity through inhibitory mechanisms that increase long-term depression of synapses (Huang et al., 2005, 2011, reviewed by Cardenas-Morales et al., 2011; however, note the previously mentioned limitations in our understanding of the physiological effects of TBS). Lesions, instead, destroy large populations of neurons as well as adjacent white matter tracts. These different consequences of TBS and lesions may also have fundamentally different effects on network activity.

Additional potential limitations of this method are that TBS may cause (a) non-specific changes that may spread across cortex and (b) that the effectiveness of TBS may vary across stimulation sites. However, we believe that neither of these two potential critiques poses strong limitations to the conclusions drawn from this study. First, we included an experimental control condition (S1) so that we could directly contrast different TBS conditions and therefore determine which effects were more likely to be due to non-specific stimulation. The widespread changes in frontal, parietal and cingulate regions were seen only after TBS to aI/fO and dlPFC, not after TBS to S1, suggesting that they were not driven by non-specific effects of stimulation. Furthermore, all of the sites in this study were over 3 cm apart, and past modeling work suggests that this should be sufficiently distant to substantially decrease the effects of strong cross-stimulation of our other target sites (Thielscher and Kammer, 2004; Lontis et al., 2006). In support of this, previous studies from our laboratory have applied TBS over more proximal lateral PFC regions and seen distinct dissociable behavioral effects (Lee et al., 2013; Blumenfeld et al., 2014). Finally, although the effectiveness of stimulation is likely to vary by stimulation site (especially in the case of aI/fO which lies further from the cortical surface), our results show that network-level changes were still seen after stimulation of this relatively deeper target. These changes are most likely driven by stimulation of the more superficial frontal operculum components of this region, rather than the deep components of the anterior insula. However, as is evident from Figure 2C the more superficial frontal operculum shows substantially the same connectivity profile as the deeper anterior insula regions and is also part of the cingulo-opercular network (as would be suggested by the joint aI/fO label). In support of this, a control analysis shows that the most superficial region along the aI/fO TMS trajectory shows significantly higher connectivity with the CO network than the FP network. Moreover, past studies have targeted similar regions of the anterior insula and frontal operculum for stimulation and successfully found predicted behavioral and neural changes (Higo et al., 2011; Lee and D'esposito, 2012). Therefore, although TBS stimulation may not be perfectly focal or reach the deepest components of the anterior insula, the method should be sufficiently precise to differentially and effectively stimulate the three target sites.

Despite the inherent limitations associated with each of these techniques, the use of combined TBS-fMRI methodology and a large within-subjects design with multiple stimulation sites allows us to greatly extend our understanding of how focal disruptions affect network communication. The results obtained in this study suggest that TBS to cognitive control regions causes widespread increases in FC across regions in frontal, parietal, and cingulate cortices. These differences suggest that the study of both acute and chronic brain perturbations may highlight unique aspects of the dynamic nature of network communication, allowing us to make more causal and temporally specific claims about interactions across these regions.

### Conflict of interest statement

The authors declare that the research was conducted in the absence of any commercial or financial relationships that could be construed as a potential conflict of interest.
